# Reference Intervals for Selected Hematology and Clinical Chemistry Measurands in Temminck's Pangolin (*Smutsia temminckii*)

**DOI:** 10.3389/fvets.2021.654529

**Published:** 2021-07-08

**Authors:** Emma H. Hooijberg, Karin Lourens, Leith C. R. Meyer

**Affiliations:** ^1^Department of Companion Animal Clinical Studies and Centre for Veterinary Wildlife Research, Faculty of Veterinary Science, University of Pretoria, Pretoria, South Africa; ^2^Department of Paraclinical Sciences and Centre for Veterinary Wildlife Research, Faculty of Veterinary Science, University of Pretoria, Pretoria, South Africa; ^3^Johannesburg Wildlife Veterinary Hospital, Johannesburg, South Africa

**Keywords:** Temminck's ground pangolin, rehabilitation, VetScan VS2, VetScan HM5, Cobas Integra 400, reference intervals, conservation medicine

## Abstract

Pangolins are the world's most trafficked non-human mammals. A significant number of Temminck's pangolins (*Smutsia temminckii*) are presented for veterinary care and rehabilitation in southern Africa. Little is known about the physiology and normal health of this species, making diagnosis and medical management difficult. This study aimed to establish reference intervals (RIs) for hematology and plasma clinical chemistry in the Temminck's pangolin. RIs were generated according to international guidelines using samples from 27 healthy free-living (*n* = 18) and rehabilitated (*n* = 9) pangolins. Hematology was performed using the Abaxis VetScan HM5 analyzer with manual differentials; clinical chemistry was performed using heparin plasma on the Abaxis VetScan VS2 and Cobas Integra 400 Plus analyzers. Hematology RIs were: RBC 3.88–8.31 × 10^12^/L, HGB 73–150 g/L, HCT 26–51%, MCV 59–72 fL, MCH 15.6–21.4 pg, MCHC 257–325 g/L, RDW 14.3–19.1%, WBC 1.80–10.71 × 10^9^/L. Vetscan VS2 clinical chemistry RIs were: albumin 27–41 g/L, ALP 26–100 U/L, ALT 25–307 U/L, amylase 267–826 U/L, bilirubin 4–10 μmol/L, calcium 2.1–2.2 mmol/L, globulin 21–55 g/L, glucose 3.8–10.0 mmol/L, phosphate 1.3–2.6 mmol/L, potassium 3.6–5.9 mmol/L, sodium 132–140 mmol/L total protein 52–84 g/L, and urea 5.3–11.4 mmol/L. RIs for creatinine were not calculated as analytical imprecision exceeded analytical performance goals. Cobas Integra clinical chemistry RIs were: albumin 22–33 g/L, ALP 20–104 U/L, ALT 17–291 U/L, amylase 466–1,533 U/L, bilirubin 1–14 μmol/L, calcium 2.0–2.4 mmol/L, creatinine <58 μmol/L, globulin 23–49 g/L, glucose 3.6–10.1 mmol/L, phosphate 1.0–2.2 mmol/L, potassium 3.1–5.8 mmol/L, sodium 137–150 mmol/L, total protein 47–72 g/L, and urea 6.0–12.5 mmol/L. There was significant bias between the two chemistry analyzers for several measurands. Differences were found for some analytes between free-living and rehabilitated animals, probably reflecting differences in nutrition and hydration. These are the first RIs generated for Temminck's pangolin. These results will allow veterinarians to better determine pangolin health status, formulate optimal treatment plans and increase patient survival rates in this endangered species.

## Introduction

Pangolins (Order: Pholidota; Family: Manidae) are myrmecophagous (eat only ants and termites), scaled, toothless mammals. Pangolins are shy, nocturnal animals, rarely encountered in the wild and difficult to keep in captivity due to their dietary requirements, lack of provision of a suitable habitat and apparent susceptibility to infectious diseases (1, 2). They are the most trafficked non-human mammal in the world, and have recently received even more notoriety as potential intermediate hosts of SARS-CoV-2 (3–5). All pangolins are listed on Appendix I of the Convention on International Trade in Endangered Species of Wild Fauna and Flora (CITES), which means that any international commercial trade of pangolins is illegal (6). These animals are nevertheless highly coveted for their meat and scales in some Asian countries, with a similar but lower demand in some African countries, and pangolin populations in China and Vietnam have been almost eliminated due to poaching. It was estimated that at least 1 million pangolins were taken from the wild in the decade preceding 2014, with a shift in trade toward African species in recent years (3, 7).

There are eight extant species of pangolins, four in Asia and four in Africa. Temminck's pangolin, *Smutsia temminckii* (other names: ground pangolin, cape pangolin, steppe pangolin) has a range from southeastern Chad down through eastern and southern Africa into South Africa (8). This pangolin species has historically had a spiritual and medicinal value to human societies across its range and has only limited value as bush-meat (8). As a result of the increase in the illegal transcontinental trade in African pangolin species in recent years, a marked increase in the number of seized live animals in Africa, all who need some level of veterinary care, has occurred (8).

Pangolins seized from the wildlife trade and presented for rehabilitation may be dehydrated and malnourished and suffer from a high burden of endo- and ectoparasites. Respiratory disease, gastric ulceration and dermatitis are common comorbidities (9). Non-trafficked animals presented to rehabilitation centers in Taiwan mainly suffered from traumatic injuries sustained from becoming caught in gin traps or holes, malnourishment, dog bite wounds, gastric ulceration and diarrhea (10). A fatal infection due to canine parvovirus-2 was recently reported in an individual free-living Taiwanese pangolin (*Manis pentadactyla pentadactyla*) (11). A novel pestivirus and a novel coltivirus were identified as causes of death in two pangolins in China; both viruses were thought to be epizootic and contracted by the animals during trafficking, and both animals showed substantial changes in clinical chemistry results before death (12). Hepatopathy and renal disease have also been described in pangolins (9). Many of these conditions are associated with significant clinical pathological changes in other species, but these have not been well-characterized in pangolins, particularly Temminck's pangolin. One of the first steps toward ensuring the utility of clinical pathology data in pangolin rehabilitation medicine, is to generate reference intervals that will aid in the interpretation of these data for rescued animals, and form the foundation of an evidence-based approach to diagnosis and treatment of pangolins.

Clinical pathology reference data from healthy individuals for two Asian pangolin species, the Sunda (*Manis javanica)* and the Chinese (*M. pentadactyla)* pangolin, and for the African white-bellied pangolin (*Phataginus tricuspis)*, have been published (13–18). Additionally, a recent study presented hematology and clinical chemistry data from 10 Temminck's pangolins (not all healthy) housed in a rehabilitation center in Zimbabwe (19). The authors of this latter paper emphasized the lack of reference intervals for this species, and the limitations this deficit places on veterinary care of Temminck's pangolins.

The aim of this study was to generate hematology and clinical chemistry reference intervals for Temminck's pangolins from South Africa. Our objectives were to include both free-living and rehabilitated animals, to use an *a priori* sampling strategy, and to design and execute the study according to American Society for Veterinary Clinical Pathology (ASVCP) guidelines for reference interval studies in veterinary species (20). We aimed to analyze the samples with commonly used point-of-care methods as well as reference laboratory methods, and we hypothesized that these analytical methods would not yield equivalent results.

## Materials and Methods

### Study Population and Sampling

The reference sample group consisted of 27 apparently healthy Temminck's pangolins. All individuals weighed more than 3 kg and were of both sexes. Animals in lactation or with a pup at foot were excluded, as were pups at foot. Eighteen of these reference individuals were free-living animals from Tswalu Kalahari Reserve (Northern Cape, South Africa) (TKR). These pangolins were tracked on foot at night and then gently caught while they were foraging and all further procedures described below were carried out at the site of capture. These animals were considered to be healthy based on observation of normal nocturnal foraging behavior and lack of any abnormalities on a clinical examination. The second group of nine pangolins were animals that had been rehabilitated after seizure from illegal trade situations and were ready for release. All of these animals were treated at the Johannesburg Wildlife Veterinary Hospital (JWVH), where they were housed in specifically designed boxes during the day and taken out in the evenings to forage for termites for 4–6 h. Rehabilitated animals were considered healthy and ready for release when the following conditions were met: (1) sufficient weight gain during the rehabilitation period and ability to successfully forage for ants and termites, (2) stable blood albumin and glucose concentrations, (3) blood smear showing no abnormalities, (4) no external wounds or outward signs of disease, and (5) no antibiotic or anti-inflammatory treatment in the preceding 2 weeks. Samples were taken from these animals on the day of release.

Pangolins roll into a tight ball as part of their defensive behavior and Temminck's pangolins cannot be unrolled while fully conscious, so blood collection is only possible when the animal is anesthetized. Both free-living and rehabilitated pangolins in this study were anesthetized by placing the curled-up animal into a modified induction chamber (a plastic container with a lid), into which a piece of cotton wool soaked in isoflurane (Isofor Inhalation Anesthetic, Piramal Healthcare, South Africa) was added. This approach achieved an acceptable level of anesthesia to facilitate working with the animals. The pangolins received isoflurane until handling was possible and were not maintained on anesthesia for the duration of the procedure. Once an animal was sufficiently anesthetised, it was taken out of the container and unrolled on a blanket in dorsal recumbency. To ensure an open airway the head was kept flexed toward the chest of the animal. Heating was supplied during winter months using a hot water bottle. Heart and respiratory rate were measured, and each animal was weighed, measured (when extended, nose to tail tip dorsally), and sexed, had ears and eyes checked, and external parasite type and load were noted. Males were identified based on the presence of inguinal testes and a penis (normally internalized, but externalized under anesthesia), females based on the presence of a vulva. Parasite load was semi-quantitatively graded on a scale of 0–5, where 0 indicated the absence of parasites, and 5 was the highest parasite load seen on an animal. The area caudal to the anus was cleaned using a skin disinfectant [F10 Skin Prep RTU, Health and Hygiene (Pty) Ltd., South Africa] so that blood could be collected from the coccygeal (tail) vein. To achieve this, a 21 G needle and a 10 mL heparinised syringe was used to collect the blood sample by inserting the needle ~1 cm caudal to the anus in the midline at a 45° downward angle in a rostral direction. A volume of 10 mL of blood was collected from each animal; 4 mL placed into a heparin tube, 4 mL into a serum tube, and 2 mL into a pediatric ethylenediamine tetraacetic acid (EDTA) tube (BD Vacutainer and BD Microtainer, Becton and Dickinson, Plymouth, United Kingdom). In addition, two blood smears were made for each animal. Once the animal had sufficiently recovered from anesthesia, it was released immediately (free-living pangolins). The time taken for data collection did not exceed 15 min and all the animals were tracked post-release to ensure that they were behaving and foraging normally. Free-living animals were marked by drilling a unique pattern of small holes into a scale to ensure no animals were sampled more than once. Animals released from JWVH all had microchips, VHF (very high frequency) and satellite trackers placed and were monitored for more than 2 months post-release to ensure they remained healthy.

### Sample Analysis

Samples collected from free-living pangolin were taken to a field research facility where they were processed, and hematological analysis was run within 3 h of collection. Serum and plasma samples were centrifuged (15,000 g for 5 min) and the plasma and serum were aliquoted and frozen (initially at −20°C at the research facility and within 5 days at −80°C) and the plasma analyzed in two batches at a later date. The serum was collected for long-term archiving. Samples collected at the JWVH were taken to the Clinical Pathology Laboratory at the Faculty of Veterinary Science in Pretoria, which is a 45-min drive away. These plasma and serum samples were centrifuged (15,000 g for 5 min) and plasma and serum aliquoted and frozen at −80°C. All plasma and serum samples were kept for more than 90 days before analysis as sample collection was done over a period of 2 years.

Samples for hematology were analyzed using the VetScan HM5 (Abaxis Europe GmbH, Griesheim, Germany) bench top analyzer which uses impedance technology for cell counting and cell volume measurement, and a cyanide-free photometric method to determine hemoglobin concentration. In a small pilot study comparing manual packed cell volume of pangolin blood, to the hematocrit (HCT) calculated by the VetScan HM5 using a variety of species settings, the bovine setting appeared to give the best match and was subsequently used. Measurands included for data analyses were the white blood cell concentration (WBC), red blood cell concentration (RBC), hemoglobin concentration (HB), HCT, mean cell volume (MCV), mean cell hemoglobin concentration (MCHC), red cell distribution width (RDW) and platelet count (PLT). One level of manufacturer-supplied quality control material was analyzed before each pangolin sample and results evaluated against the manufacturer's target ranges.

Blood smears were stained with a Wright-Giemsa stain on an automated stainer (HemaTek 2000, Siemens Healthcare, Erlangen, Germany). A 200-cell manual leukocyte differential count was performed by a board-certified clinical pathologist (EHH). Observations on morphological characteristics of the erythron, leukon, and thrombon were recorded.

Clinical chemistry analysis was performed on two analyzers (on the same day): the Cobas Integra 400 Plus [Roche Products (Pty) Ltd., Basel, Switzerland] and the Vetscan VS2 (Abaxis Europe GmbH, Griesheim, Germany). The Cobas Integra is an automated wet chemistry analyzer common in medium-throughput veterinary laboratories. Daily internal and monthly external quality control was performed according to laboratory protocols. The Vetscan VS2 is a portable benchtop analyzer, which uses pre-packaged rotors with liquid reagents. The Comprehensive Diagnostic Profile rotor was used in our study. An internal quality control procedure is run on each rotor before each analysis; no quality control material is supplied by the manufacturer. The measurands included in the rotor and analytical methods for both analyzers are presented in Table 1. Plasma samples were thawed overnight, brought to room temperature and gently vortexed to promote homogenization before analysis.

**Table 1:** Analytical methods utilized by the Abaxis Vetscan VS2 and Roche Cobas Integra 400 Plus for the analysis of Temminck's pangolin heparin plasma samples.

	**Roche Cobas Integra 400 Plus**	**Abaxis Vetscan VS2**
Albumin	Bromocresol green dye-binding method	As for Cobas Integra
ALT	Kinetic (L-alanine and 2-oxyglutarate)	As for Cobas Integra
ALP	Kinetic (p-nitrophenol phosphate)	As for Cobas Integra
Amylase	Enzymatic (ethylidene-G7PNP)	Enzymatic (2-chloro-p-nitrophenyl-D-maltotrioside)
Bilirubin	Diazo Method	Enzymatic (bilirubin oxidase)
Calcium	Cresophthalein complexone method	Arsenazo III method
Creatinine	Modified Jaffe reaction	Enzymatic (creatinine amidohydrolase)
Globulin	Calculated	Calculated
Glucose	Hexokinase method	As for Cobas Integra
Phosphate	Phosphomolybdate method	Enzymatic (glucose-6-phosphate dehydrogenase)
Potassium	Ion-selective electrode	Enzymatic (pyruvate kinase)
Sodium	Ion-selective electrode	Enzymatic (beta-galactosidase)
Total protein	Biuret Method	As for Cobas Integra
Urea	Enzymatic (urease)	As for Cobas Integra

*ALP, alkaline phosphatase; ALT, alanine aminotransferase*.

### Clinical Chemistry Imprecision and Method Comparison

None of the analyzers used in this study have been validated for use in pangolin. A full method validation study was not possible due to limited resources. In order to obtain at least an estimate of short-term imprecision specifically for pangolin plasma, remaining specimen material was pooled and measured 15 times within one run on the Cobas Integra and 12 times in 1 day (using 12 rotors) on the Vetscan VS2. Imprecision, represented by the coefficient of variation (CV%), was calculated from the mean and standard deviation (SD) of the results. The CV for each measurand for each analyzer was compared to total allowable error (TE_a_) recommended for veterinary species, and imprecision was determined to be unacceptable if the CV exceeded the TE_a_ (21). For those measurands where this was the case, no further data analyses were performed.

Previous publications have demonstrated that a significant bias may exist between the analytical methods used by the Cobas and the Vetscan VS2 for several species (22, 23). Although sample sizes in this study were low, routine method comparison statistics were applied in order to obtain and evaluate estimates of bias between the two analyzers for pangolin plasma (24, 25). Data were analyzed using Spearman's correlation coefficients, a CUSUM test for linearity, Passing-Bablok regression analysis and Bland-Altman difference plots. Using the equation for the regression line, a significant constant bias was considered to be present if the 95% confidence intervals (CI) around the y-intercept did not include zero; a significant proportional bias was present if the 95% CI around the slope did not include 1.0. The mean percentage bias, derived from the Bland-Altman plots, was considered unacceptable if it exceeded TE_a_. These statistics were not applied to globulin concentration, as this measurand is calculated.

### Reference Intervals

Reference intervals were generated according to ASVCP guidelines (20). Firstly, data were examined visually using histograms and descriptive statistics were generated. Dixon and Tukey tests were used to identify outliers and if any data point was identified as an outlier, that data point was excluded, and the statistical analysis rerun. The same data points were excluded for both chemistry analysers. This procedure was repeated until no more outliers remained, for any measurands.

The Anderson-Darling test was used to assess whether the data had a Gaussian distribution; in order to increase specificity for the small data set, a *p*-value of < 0.27 was considered significant (26). The runs test was used to evaluate the symmetry of the data (*p* < 0.05). Reference intervals were calculated as follows (20): If untransformed data had a Gaussian distribution, then the 95% reference intervals were calculated using the parametric method. Non-Gaussian data were Box-Cox transformed, and the robust method was used to calculate the reference intervals, if data were also symmetrical. The 90% confidence interval of the lower and upper reference limits was calculated with a bootstrap method. If data were not Gaussian and symmetrical after transformation, then reference intervals were calculated using the non-parametric method. Using the latter method for sample sizes with <40 individuals means that the minimum and maximum values become the reference limits, and that the 90% CIs of these cannot be calculated.

Although sample size was not large enough for partitioning, results from free-living and rehabilitated pangolins were compared using the Mann-Whitney U test (*p* < 0.05) (20).

Statistical analyses were performed with MedCalc software version 19.1.7 (MedCalc Software, Ostend, Belgium) and the Excel add-on Reference Value (RefVal) Advisor version 2.1 (27). Ethical approval for this study was obtained from the University of Pretoria Animal Ethics Committee (V069-17).

## Results

All original data can be viewed in Supplementary Table 1.

### Study Population and Sample Analysis

Twenty-seven pangolins (18 free-living animals and nine rehabilitated animals) were included in the reference sample population. Pangolins ranged in weight from 3.4 to 15.1 kg and in length (nose to tail tip) from 53 to 114 cm. It has been suggested that Temminck's pangolins weighing <6 kg are juvenile while those weighing >6 kg are adult (2). Five animals in the reference sample population weighed <6 kg. There were 11 males and 16 females. All animals appeared to be healthy. Twenty-three of the 27 animals were checked for ectoparasites, of which 15 had parasites (ticks or mites or both) and eight did not (score of 0). Of these 15, five had a low parasite load (score of 1), six had a medium load (score of 2–3) and four had a high load (score of 4–5). Of the TKR animals sampled, 64% (8/14) had parasites and of the JWVH animals sampled 78% (7/9) had parasites.

Plasma samples from all 27 animals were assayed for clinical chemistry, but hematology was only performed on samples from 25 animals as blood from two rehabilitated individuals could not be analyzed within 24 h. Moreover, blood smears from only 23 animals were examined as smears from a further two animals were damaged during transport. No hemolysis, icterus, or lipemia was present in any of the plasma samples. Results from quality control runs were within laboratory performance limits, manufacturer target limits, or passed an electronic quality control check, as applicable.

### Clinical Chemistry Imprecision and Method Comparison

Results of the short-term imprecision experiment are presented in Table 2. Coefficients of variation were less than TE_a_ for all measurands, apart from creatinine on the VetScan VS2 (26.5%). Creatinine results from one pooled sample ranged from <18 to 51 μmol/L. As this analytical error could have influenced results from the reference individuals, reference intervals for VetScan VS2 creatinine were not created.

**Table 2:** Short-term imprecision (CV), Spearman's correlation coefficient (*r*), Passing-Bablok regression data (intercept and slope), and mean bias of the Abaxis Vetscan VS2 compared to the Roche Cobas Integra 400 Plus for Temminck's pangolin heparin plasma.

**Analyte**	**CV_**VS2**_ (%)**	**CV_**COBAS**_ (%)**	***r***	**Intercept (95% CI)**	**Slope (95% CI)**	**Mean bias**	**Mean bias (%)**	**TE_**a**_**
Albumin	1.9	1.2	0.74	−7.00 (−14.00 to 0.00)	1.50 (1.25 to 0.75)^b^	7.4 g/L	23.8^c^	15%
ALP	5.1	1.2	0.97	5.18 (0.29 to 11.13)^a^	0.96 (0.87 to 1.03)	−3 U/L	−3.4	25%
ALT	1.3	1.3	0.99	9.81 (7.00 to 11.07)^a^	0.96 (0.93 to 1.00)	7.7 U/L	14.9	25%
Amylase	1.1	1.0	0.98	6.46 (−28.88 to 7.02)	0.53 (0.50 to 0.57)^b^	−466 U/L	−58.0^c^	30%
Bilirubin	7.2	14.9	0.31	3.21 (1.39 to 5.30)^a^	0.6 (0.15 to 0.98)^b^	1 μmol/L	28.6^c^	20%
Calcium	1.3	0.4	0.67	0.1 (−1.00 to 0.1)	1.00 (1.00 to 1.50)	0.11 mmol/L	4.9	20%
Creatinine	26.5^c^	3.0	Not done					20%
Glucose	1.5	0.7	0.99	0.35 (0.00 to 0.66)	0.94 (0.89 to 1.00)	−0.01 mmol/L	−6.4	20%
Phosphorus	1.7	0.7	0.96	0.50 (0.08 to 0.50)^a^	1.00 (1.00 to 1.30)	0.48 mmol/L	31.0^c^	15%
Potassium	3.2	0.7	0.95	0.94 (0.70 to 1.59)^a^	0.93 (0.77 to 1.00)	0.64 mmol/L	3.8	5%
Sodium	0.9	0.2	0.52	40.67 (−7.00 to 72.78)	0.67 (0.44 to 1.00)	−7 mmol/L	5.0	5%
Total protein	1.2	0.4	0.94	−2.00 (−13.67 to 7.00)	1.14 (1.00 to 1.30)	6.9 g/L	10.5^c^	10%
Urea	0.8	1.2	0.99	−0.53 (−1.00 to −0.02)^a^	0.95 (0.92 to 1.00)	−0.93 mmol/L	−9.9	12%

*Estimates of mean bias are derived from Bland-Altman difference plots*.

a *The 95% confidence interval of the y-intercept does not include zero: a constant bias is present*.

b *The 95% confidence interval of the slope does not include 1.0: a proportional bias is present*.

c *CV or bias (%) greater than total allowable error*.

*CV, coefficient of variation; CI, confidence interval; TE_a_, total allowable error, ALP, alkaline phosphatase; ALT, alanine aminotransferase*.

Either a significant constant or proportional bias, or a bias exceeding TE_a_, was evident when the Vetscan VS2 was compared to the Cobas Integra for all measurands except calcium, glucose and sodium. Results are displayed in Table 2.

### Hematology Reference Intervals and Blood Smear Examination

**Figure 1:**
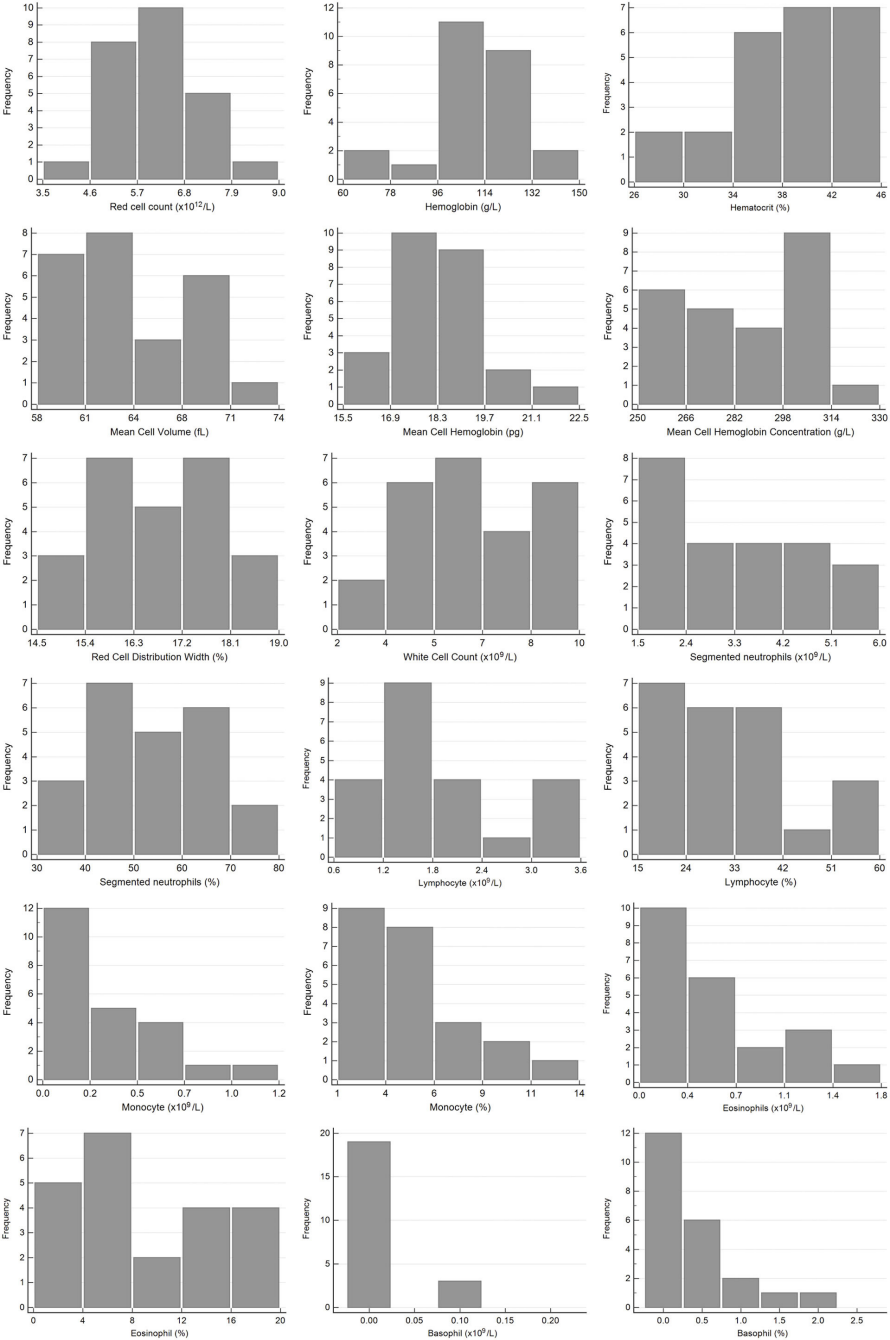
Histograms showing the distribution of results for hematology measurands from Temminck's pangolin for the Abaxis Vetscan HM5 and manual differential leukocyte counts. The x-axis represents the measurand result; the y-axis represents the frequency of these values occurring.

Hematology reference intervals are presented in Table 3, with corresponding histograms shown in Figure 1. Reference intervals were not generated for platelets due to the high prevalence of clumping. Five of the samples had platelet counts of <100 × 10^9^/L. Numerous small platelet clumps were present on most of the blood smears, including from these five samples, and the platelet counts were considered inaccurate. The automated platelet counts ranged from 16 to 318 × 10^9^/L with a median of 197 × 10^9^/L. One high outlier each for HCT, absolute lymphocyte count (both from one individual), absolute and relative eosinophil count (one individual) and absolute and relative basophil count (one individual) were identified and excluded.

**Table 3:** Hematology reference intervals for Temminck's pangolin using the Abaxis HM5 hematology analyser and 200-cell manual leukocyte differential counts.

**Measurand**	***n***	**Mean**	**Median**	**SD**	**Min**	**Max**	**RI**	**LRL 90%CI**	**URL 90% CI**	**Distribution**	**Method**
RBC (×10^12^/L)	25	6.10	6.22	1.05	3.78	8.48	3.88–8.31	3.33–4.58^a^	7.62–8.84^a^	G	P
HGB (g/L)	25	111	111	18	66	146	73–150	62–84^a^	139–160^a^	G	P
HCT (%)	24	38	39	5	26	45	26–45	Not computed		NG	NP
MCV (fL)	25	64	63	4	59	72	59–72	Not computed		NG	NP
MCH (pg)	25	18.3	18.1	1.4	15.8	21.8	15.6–21.4	15.1–16.4^a^	20.4–22.5^a^	NG	T, R
MCHC (g/L)	25	287	291	21	257	325	257–325	Not computed		NG	NP
RDW (%)	25	16.7	16.8	1.1	14.7	18.5	14.3–19.1	13.7–15.0^a^	18.4–19.7^a^	G	P
WBC (×10^9^/L)	25	6.25	6.33	2.12	2.81	9.63	1.80–10.71	0.68–3.00^a^	9.44–11.92^a^	G	P
Neutrophil (×10^9^/L)	23	3.33	3.22	1.32	1.59	5.61	1.19–7.04	0.90–1.62	5.83–8.26^a^	NG	T, R
Neutrophil (%)	23	53.0	50.0	12.0	33.0	77.0	27.0–79.0	20.0–35.0^a^	72.0–87.0^a^	G	P
Lymphocyte (×10^9^/L)	22	1.83	1.54	0.81	0.71	3.40	0.64–4.35	0.51–0.83	3.16–5.50^a^	NG	T, R
Lymphocyte (%)	23	31.0	30.0	12.0	15.0	57.0	13.0–63.0	11.0–16.0	52.0–74.0^a^	NG	T, R
Monocyte (×10^9^/L)	23	0.31	0.18	0.25	0.03	1.02	0.03–1.26	0.02–0.06	0.72–1.80^a^	NG	T, R
Monocyte (%)	23	5.0	4.0	3.0	1.0	14.0	1.0–14.0	0.0–1.0	11.0–18.0^a^	NG	T, R
Eosinophil (×10^9^/L)	22	0.56	0.49	0.43	0.05	1.55	0.01–1.95	0.00–0.07	1.36–2.71^a^	NG	T, P
Eosinophil (%)	22	8.9	6.8	5.9	1.0	18.5	0.3–27.0	0.0–1.4	20.0–33.9^a^	NG	T, R
Basophils (×10^9^/L)	22	0.39	0.00	0.	0.00	0.34	0.00–0.34	Not computed		NG	NP
Basophil (%)	22	0.4	0.0	0.6	0.0	2.0	0.0–2.0	Not computed		NG	NP

*Where the non-parametric method was used, reference limits could not be calculated due to the small sample size, and the minimum-maximum range represents the reference interval*.

a *The ratio of the CI to the RI exceeds 0.2 (20%)*.

*n, number of reference individuals; SD, standard deviation; RI, reference interval; LRL, lower reference limit; URL, upper reference limit; CI, confidence interval; G, Gaussian; NG, non-Gaussian; P, parametric; R, robust; T, Box-Cox transformed; RBC, red blood cell concentration; HGB, hemoglobin concentration; HCT, hematocrit; MCV, mean cell volume; MCH, mean cell hemoglobin; MCHC, mean cell hemoglobin concentration; RDW, red cell distribution width; WBC, white blood cell concentration*.

**Figure 2:**
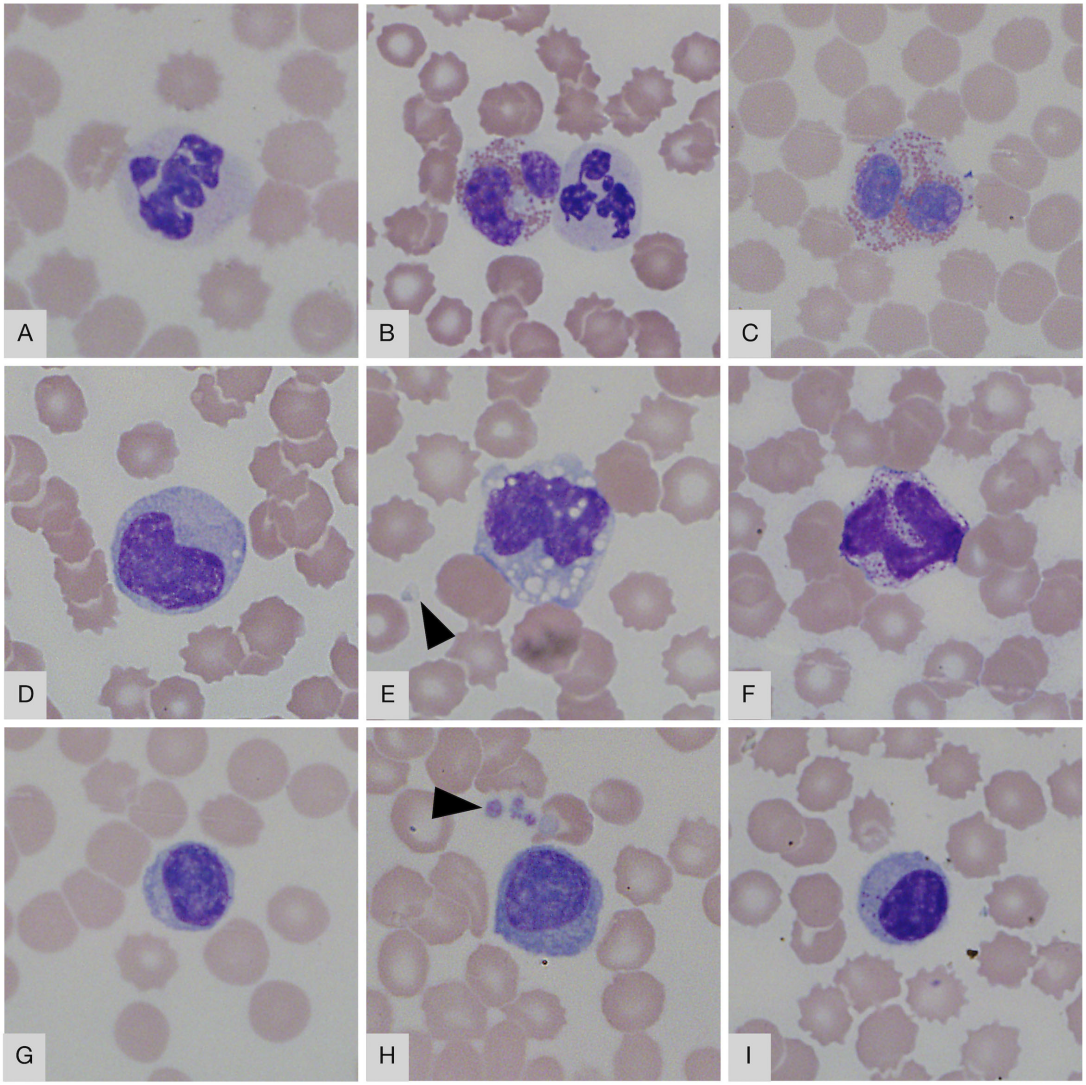
Blood smear images from Temminck's ground pangolin. **(A)** neutrophil; **(B)** neutrophil (right) and eosinophil (left); **(C)** eosinophil; **(D)** monocyte; **(E)** monocyte with vacuoles; **(F)** basophil; **(G)** small lymphocyte; **(H)** reactive lymphocyte; **(I)** granular lymphocyte. Platelets are visible in **(E,H)** (arrowheads). Wright-Giemsa, × 1,000 objective.

In Wright-Giemsa-stained films, the erythrocytes had a diameter of 7–9 μm and were round in shape with a mild central pallor or lacked central pallor (Figure 2). Some of the smears showed mild rouleaux formation. Occasional schistocytes and acanthocytes were seen (<1/1,000 × high power field). Many crenated erythrocytes were present, presumably due to a preparation artifact. Intra-erythrocytic piroplasms were present (ranging from rare to a few) in six smears (five free-living, one rehabilitated). These could not be identified based on their morphology, and further molecular diagnostic investigation is currently underway. None of the pangolins with parasites were individuals with outliers. The neutrophils were round (diameter 12–15 μm) with a moderate amount of clear to light blue cytoplasm containing fine, pale pink granules and segmented nuclei with 2–5 lobes with a coarse chromatin pattern (Figures 2A,B). Eosinophils were round and larger than neutrophils (diameter 16–17 μm) with a moderate amount of clear to light blue cytoplasm with prominent small, round, orange-pink granules. The nuclei were segmented with two lobes and a coarse chromatin pattern (Figures 2B,C). Basophils were round (diameter 13–15 μm) with a moderate amount of clear cytoplasm containing numerous purple granules. The nuclei had two lobes with finely stippled chromatin (Figure 2F). Monocytes had round to pleomorphic shapes (diameter 14–18 μm) with moderate to abundant light blue-gray cytoplasm. Cytoplasmic vacuoles were occasionally present. Monocyte nuclei were irregularly round, oval, or bean-shaped, with finely stippled chromatin (Figures 2D,E). The lymphocytes were round in shape (diameter 9–11 μm) with round to slightly oval nuclei. They had a scant amount of blue cytoplasm with dense- to coarsely clumped cytoplasm (Figure 2G). A few reactive lymphocytes were also noted: these were 1.5 times larger, with an increased amount of cytoplasm, sometimes darker blue (Figure 2H). Occasional lymphocytes with this morphology also had magenta-staining cytoplasmic granules, consistent with a large granular lymphocyte morphology (Figure 2I). Platelets were round or oval in shape with occasionally very elongated forms (diameter 2–5 μm). The cytoplasm was pale pink with centrally located purple granules. Small to large platelet aggregates were seen on most smears (Figures 2E,H).

### Clinical Chemistry Reference Intervals

**Figure 3:**
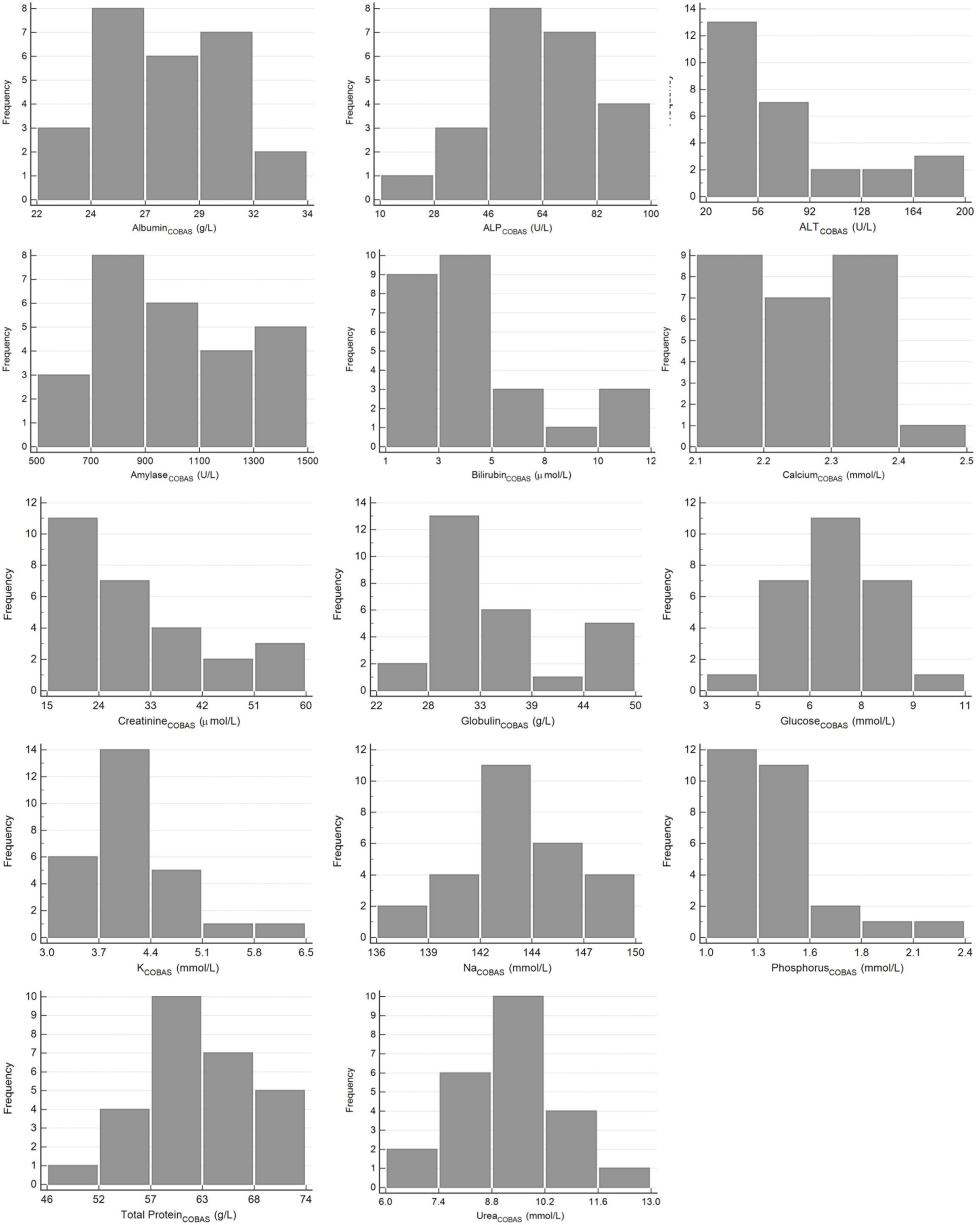
Histograms showing the distribution of results for plasma clinical chemistry measurands from Temminck's pangolin for the Roche Cobas Integra 400 Plus. The x-axis represents the measurand result; the y-axis represents the frequency of these values occurring.

**Figure 4:**
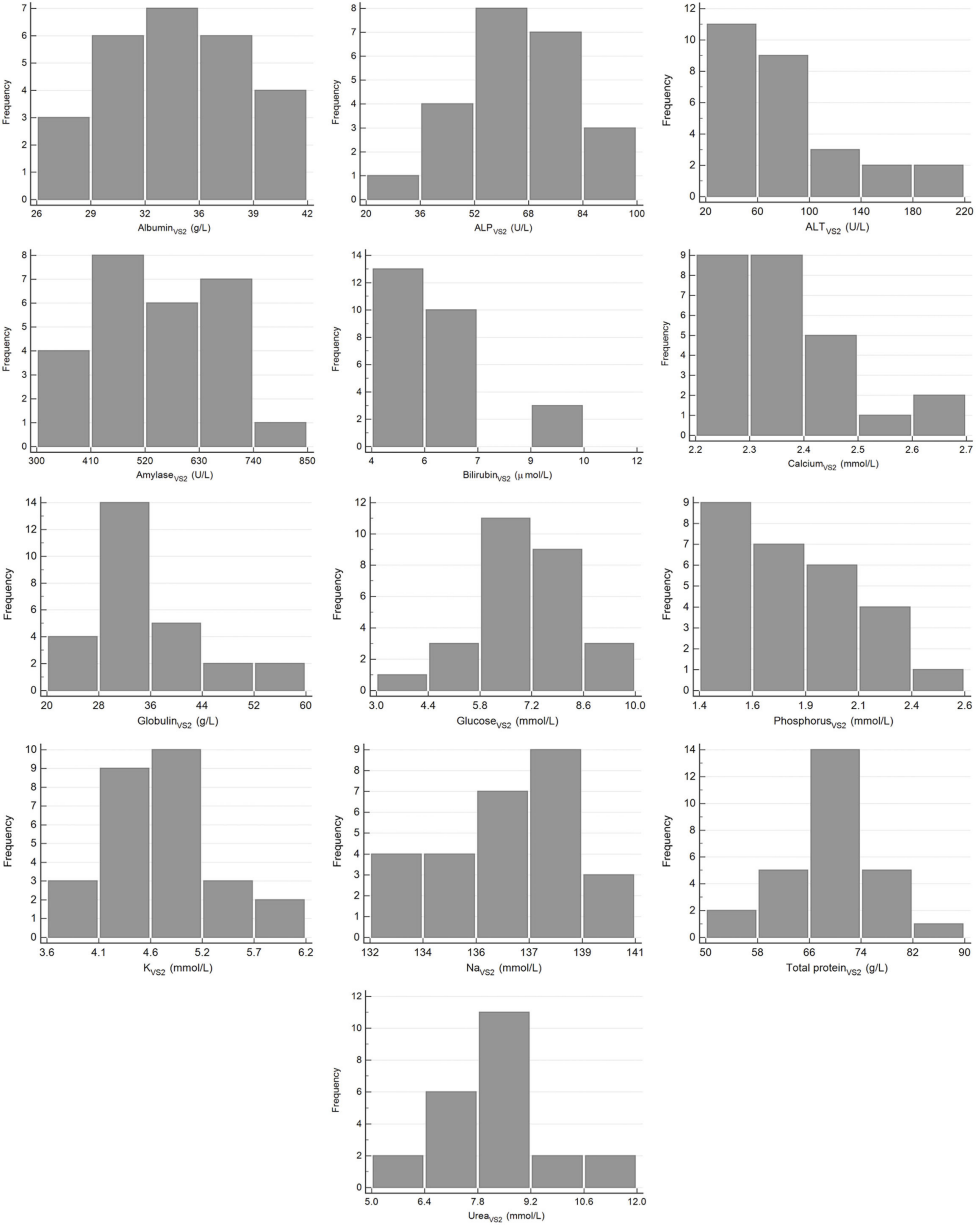
Histograms showing the distribution of results for plasma clinical chemistry measurands from Temminck's pangolin for the Abaxis Vetscan VS2. The x-axis represents the measurand result; the y-axis represents the frequency of these values occurring.

The results for reference intervals generated for plasma clinical chemistry measurands for the Cobas Integra and VetScan VS2 are presented in Table 4, and histograms are presented in Figures 3, 4. One low outlier each for albumin and calcium, one high outlier each for amylase and bilirubin and four high outliers for ALP and urea were identified and excluded. One rehabilitated pangolin had two outliers (amylase, urea), one pangolin from TKR had three outliers [albumin, alkaline phosphatase (ALP), urea]. The other seven outliers were individual results in seven other pangolins. For creatinine on the Cobas Integra, four results were below the limit of detection (18 μmol/L), and the reference interval is given as less than the maximum value (<58 μmol/L).

**Table 4:** Clinical chemistry reference intervals for Temminck's pangolin measured from heparin plasma on the Abaxis VetScan VS2 and Roche Cobas Integra 400 Plus analyzers.

**Measurand**	***n***	**Mean**	**Median**	**SD**	**Minimum**	**Maximum**	**RI**	**LRL 90% CI**	**URL 90% CI**	**Distribution**	**Method**
**Albumin (g/L)**											
Cobas	26	27	27	3	22	32	22–33	20–23^a^	32–35^a^	NG	T, R
VetScan	26	34	34	4	27	41	27–41	Not computed		NG	NP
**ALP (U/L)**											
Cobas	23	62	55	20	19	95	20–104	9–32^a^	91–116^a^	G	P
VetScan	23	63	60	17	23	95	26–100	16–36^a^	89–110^a^	G	P
**ALT (U/L)**											
Cobas	27	74	59	51	22	192	17–291	14–23	173–464^a^	NG	T, R
VetScan	27	82	72	50	31	198	25–307	21–31	184–475^a^	NG	T, R
**Amylase (U/L)**											
Cobas	26	999	978	254	533	1,441	466–1,533	334–607^a^	1,384–1,675^a^	G	P
VetScan	26	546	528	133	317	788	267–826	197–341^a^	748–901^a^	G	P
**Bilirubin (μmol/L)**											
Cobas	26	5	4	3	1	11	1–14	1–2	9–20^a^	NG	T, R
VetScan	26	6	6	2	4	10	4–10	Not computed		NG	NP
**Calcium (mmol/L)**											
Cobas	26	2.2	2.2	0.1	2.1	2.4	2.0–2.4	2.0–2.1^a^	2.3–2.4^a^	G	P
VetScan	26	2.3	2.3	0.1	2.2	2.6	2.1–2.6	2.1–2.2^a^	2.5–2.8^a^	NG	T, R
**Creatinine (μmol/L)**											
Cobas	27	31	31	12	<18	58	4 results under detection limit, <58 μmol/L used as RI				
VetScan	Not done: imprecision exceeds total allowable error										
**Globulin (g/L)**											
Cobas	27	35	33	7	23	49	23–49	Not computed		NG	NP
VetScan	27	34	32	9	21	55	21–55	Not computed		NG	NP
**Glucose (mmol/L)**											
Cobas	27	6.9	6.7	1.6	3.0	9.8	3.6–10.1	2.8–4.5^a^	9.2–11.0^a^	G	P
VetScan	27	6.9	6.8	1.5	3.1	9.4	3.8–10.0	2.9–4.6^a^	9.1–10.8^a^	G	P
**Phosphate (mmol/L)**											
Cobas	27	1.3	1.3	0.3	1.0	2.2	1.0–2.2	Not computed		NG	NP
VetScan	27	1.8	1.7	0.3	1.4	2.5	1.3–2.6	1.3–1.4	2.3–2.9^a^	NG	T, R
**Potassium (mmol/L)**											
Cobas	27	4.1	4.1	0.6	3.0	6.0	3.1–5.8	2.9–3.5	5.2–6.5^a^	NG	T, R
VetScan	27	4.8	4.7	0.5	3.7	6.0	3.6–5.9	3.3–3.9^a^	5.6–6.2	G	P
**Sodium (mmol/L)**											
Cobas	27	144	144	3	137	149	137–150	135–139^a^	149–152^a^	G	P
VetScan	27	137	137	2	132	140	132–140	Not computed		NG	NP
**Total protein (g/L)**											
Cobas	27	62	62	6	47	72	47–72	Not computed		NG	NP
VetScan	27	69	68	8	52	84	52–84	Not computed		NG	NP
**Urea (mmol/L)**											
Cobas	23	9.3	9.4	1.5	6.0	12.4	6.0–12.5	5.2–6.9	11.6–13.5	G	P
VetScan	23	8.4	8.3	1.4	5.2	11.6	5.3–11.4	4.5–6.2^a^	10.5–12.3^a^	G	P

*Where the non-parametric method was used, reference limits could not be calculated due to the small sample size, and the minimum-maximum range represents the reference interval*.

a *The ratio of the CI to the RI exceeds 0.2 (20%)*.

*ALP, alkaline phosphatase; ALT, alanine aminotransferase; n, number of individuals; SD, standard deviation, RI, reference interval, CI, confidence interval; LRL, lower reference limit; URL, upper reference limit; G, Gaussian; NG, non-Gaussian; P, parametric; NP, non-parametric; R, robust method; T, Box-Cox transformation*.

### Differences Between Free-Living and Rehabilitated Animals

Hematocrit, HB, RBC, globulins, total protein and sodium (VetScan only) were significantly higher and lymphocyte count, ALT, amylase, bilirubin (Cobas only), and potassium (VetScan only) were significantly lower in the free-living compared to the rehabilitated pangolins. Median and range for these measurands for the two groups, as well as the *p*-values, are presented in Table 5.

**Table 5:** Median and range (in parentheses) for measurands that were significantly different between free-living and rehabilitated Temminck's pangolins.

**Measurand**	**Free-living**	**Rehabilitated**	***p-*value**
RBC (×10^12^/L)	6.5 (5.3–7.4)	4.8 (3.8–8.5)	0.0101
HGB (g/L)	119 98–132)	100 (66–146)	0.0251
HCT (%)	41 (35–45)	32 (26–37)	0.0007
Lymphocyte (×10^9^/L)	1.7 (0.7–3.4)	1.2 (0.8–2.0)	0.0412
ALT (U/L) (Cobas)	44 (22–158)	100 (39–192)	0.0036
ALT (U/L) (VetScan)	53 (31–166)	106 (43–198)	0.0069
Amylase (U/L) Cobas	881 (533–1,338)	1,219 (847–1,442)	0.0124
Amylase (U/L) Vetscan	480 (317–722)	634 (459–788)	0.0302
Bilirubin (μmol/L) (Cobas)	3.2 (1.3–10.4)	6.6 (3.3–11.0)	0.0029
Globulin (g/L) Cobas	35 (30–49)	31 (23–34)	0.0018
Globulin (g/L) VetScan	35 (25–55)	30 (21–37)	0.0203
Potassium (mmol/L) VetScan	4.6 (3.7–6.0)	5.0 (4.3–6.0)	0.0230
Sodium (mmol/L) VetScan	138 (133–140)	135 (132–137)	0.0042
Total protein (g/L) Cobas	63 (59–72)	56 (47–61)	0.0001
Total protein (g/L) VetScan	70 (62–84)	62 (52–68)	0.0006

*The p-value for the Mann-Whitney-test is included*.

## Discussion

This study reports reference intervals, generated according to ASVCP guidelines, for hematology and clinical chemistry derived from a small reference sample group of both free-living and rehabilitated Temminck's pangolin in South Africa. These reference intervals will assist in the health assessment and treatment of individual pangolins, particularly those confiscated from the illegal wildlife trade.

A moderate approach to outlier analysis was applied here, in which all single results identified by outlier analysis were removed from the data set but remaining non-outlier data from that individual were kept. Outliers represent erroneous results stemming from errors that may have occurred in any of the phases of the study, and retention of outliers can result in reference intervals that are inappropriately wide and not clinically useful (28). Determining the health status of non-domestic species is challenging, and the detailed investigations routinely used to confirm health in domestic animals are often not possible. In this study, lack of disease in the free-living pangolins was assumed based on the observation of normal nocturnal foraging behavior and lack of obvious abnormalities on clinical examination. In the rehabilitated animals, the inclusion criteria were more stringent. It is possible that animals in either group were in fact not completely healthy, and so outlying results were eliminated in case they represented clinical pathology changes relating to a diseased state (29).

A sample size of at least 120 reference individuals is recommended in order to ensure accuracy of the 95% population reference limits (20). The sample size of 27 pangolins is small, with results from as few as 22 individuals used to generate reference intervals for some measurands, after outlier exclusion. The number of reference individuals sampled was limited by the rarity of this species and the difficulty in finding them in their natural environment. It has been recognized that sample size is often limited when collecting reference data from rare species, but that the reference intervals generated using recommended methods is still valuable (19). The accuracy of the reference limits, as represented by the CI around the limits, is lower with smaller sample sizes. This was evident for most measurands here, as the CI to reference interval ratio was >0.2 for at least one of the reference limits, for most measurands (20). Results from a recent modeling study have suggested that using the non-parametric method only, and not the methods recommended by the ASVCP, may be more accurate when *n* = 20 (30). Using the non-parametric method with such a small sample size effectively means that the minimum and maximum values become the lower and upper reference limits. The minimum and maximum values are presented in Tables 3, 4 here for all measurands, and clinicians are advised to use both the minimum-maximum range and the reference interval for interpretation of results from pangolin patients, based on these recent findings.

The VetScan VS2 has featured in several reference interval studies, covering species ranging from domestic cats, to owls, prairie dogs and Sunda pangolins, to name just a few (13, 18, 22, 23, 31). This analyzer is a portable point-of-care device and requires a sample volume of only 0.1 mL, which makes it popular with veterinarians working with non-domestic species. Blood samples from wildlife may also be submitted to reference laboratories, which have analyzers using different analytical methods. Several studies have demonstrated significant differences between results obtained from the VetScan VS2 and reference laboratory methods (23, 31–34). Bias between the Vetscan VS2 and Cobas Integra was also found for most measurands, although the sample number and range of results used for the statistical analyses was lower than recommended (24, 35). The demonstration of a bias between two methods necessitates the generation of reference intervals for each method (22, 32, 36), which was subsequently performed here. Ideally, the analytical methods used for reference interval studies should be validated for the species in question (20, 37). This validation is often not possible for non-domestic species, due to a paucity of sample material, but estimates of analytical error for each method (imprecision, bias, total observed analytical error) should at least be known and fall within predetermined performance goals for methods used to generate data for reference interval studies (20). Although internal quality control results were acceptable on both clinical chemistry analyzers, imprecision for creatinine determined using pangolin plasma on the VetScan VS2 was not acceptable. The reason for this imprecision is unclear. Imprecision for measurands measured by the same lamp (bilirubin, sodium and total protein) were acceptable, and imprecision for creatinine on the Cobas Integra was low, making a species-specific matrix effect unlikely. Assessment of VetScan VS2 creatinine imprecision with a different sample, for example liquid quality control material or canine plasma, was not performed as part of this study. The high analytical imprecision may have affected creatinine data from the reference individuals, and reference intervals were not calculated.

The hematology analyzer used in this study was not validated for Temminck's pangolins. Based on internal laboratory results, the bovine setting appeared to be the most appropriate for this species, however manual packed cell volumes were not performed in the field and so the accuracy of this choice is not known. Manual determination of packed cell volume is considered to be the gold standard method, compared to a HCT calculated by an analyzer, and the lack of packed cell volume data is a limitation of this study (35). The reference intervals generated for hematology are therefore not only specific to the VetScan HM5, but also specific to the use of the bovine setting on this analyzer.

In terms of published reference data for hematology in pangolins, the two studies involving Sunda pangolins had the most similar design to ours, with analysis performed on a VetScan HM5 using the canine setting (13, 18). Fifty-one rescued Sunda pangolins, with no evidence of disease on clinical examination, were included in the first study, and 13 healthy captive pangolins in the second study. While reference data and intervals from the Sunda pangolins are generally similar to those calculated here for Temminck's pangolins, they are often wider. This is particularly true for the study with 13 captive individuals, where ranges and standard deviations for some measurands are of a very high magnitude, and not suitable for comparison to our data, due to the small sample size and lack of outlier exclusion in that study (18). All individuals in the larger rescued Sunda pangolin group were sampled within 24 h of rescue, and were presumably in a physiologic state of stress, which may have caused a greater variation in results. For example, reference intervals in Sunda pangolins are slightly higher for neutrophils and monocytes, and lower for lymphocytes which probably represent a tendency toward a stress leukogram in those animals. Although not directly comparable, as different hematology analyzers were used, most reference intervals presented for Chinese pangolins in two different studies are similar to those for Temminck's pangolin, although also wider (14, 15). Mean values derived from hematology analysis on two different analyzers for eight Temminck's pangolins in a rehabilitation center in Zimbabwe are also mostly similar to ours (19). The hematology reference intervals which differ in our study to all other published data, are those for eosinophils and basophils, with a higher upper reference limit. All pangolins with an eosinophil percentage >12%, and two of four animals with a basophil percentage ≥1% (Figure 1), were free-living animals. Both endo- and ectoparasites have been implicated as a cause of eosinophilia and basophilia (38). Spiruroid nematodes, mites, and a range of tick species have been reported to occur in Temminck's pangolin (39). The majority of the pangolins sampled for this study had ectoparasites, although it was the animals in the rehabilitated group, not the free-living pangolins, that were more commonly affected. This finding could be attributed to the fact that these animals were all confiscated from illegal traders, where they are often kept in less than ideal conditions. They are thus stressed and unable to groom themselves, which may contribute to the higher prevalence of ectoparasites on these animals, compared to the free-living population. The ectoparasite burden in the animals with a relatively high proportion of eosinophils ranged from low to high and three animals with a basophil percentage ≥1% did not have appreciable ectoparasite loads (data for the fourth animal were not collected). There does not seem to be an obvious association between increased eosinophils and basophils in some of these animals, and the reason for these findings is unknown.

Blood cell morphology has not been systematically documented in any pangolin species but was similar here to that described for carnivores. A case report describing hematology and clinical chemistry in a sick Indian pangolin (*M. crassicaudata*) presents some cytomorphometric values for erythrocytes and selected leukocytes, which are similar to our findings, apart from that the eosinophils and monocytes from that pangolin were smaller than those of Temminck's pangolin (40). Blood parasites, like *Theileria* and *Babesia* spp., are commonly observed in African wildlife, and are often considered to be incidental findings, with host and parasite existing in a state of endemic stability (41). The presence of a hemoparasite in this state may not influence the hemogram (42). The six pangolins with intra-erythrocytic piroplasms in this study did not have hematological measurands that were identified as outliers, and, as there did not seem to be an effect of the parasite on hematology, these animals were not excluded.

Noteworthy differences are present for some clinical chemistry measurands, between the Asian pangolins and Temminck's pangolin, even taking differences in analytical methods into account. The Sunda pangolin studies also used the VetScan VS2, so results are directly comparable (13, 18). The two Chinese pangolin studies used a dry slide and a wet chemistry analyzer (14, 15). The creatinine concentrations (Cobas Integra) found in Temminck's pangolin were much lower than those reported in one of the Chinese pangolin studies and the Sunda pangolin studies and are similar to the data reported for this species in Zimbabwe (13–15, 18, 19). Creatinine concentrations are dependent on muscle mass and glomerular filtration rate (43). The Chinese pangolins, for example, ranged in weight from 1.5 to 7.7 kg compared to Temminck's pangolin that ranged from 3.4 to 15.1 kg (14). Temminck's pangolins are larger than Chinese pangolins, with a presumably higher muscle mass, but unexpectedly lower creatinine concentrations. This difference could be due to a disparity in muscling, or hydration status, between species and individuals involved in these studies. Temminck's pangolins are apparently water-independent and may have different renal physiology (for example, a higher glomerular filtration rate) to Asian species (8).

Reference intervals for albumin, total protein and urea were lower in Temminck's pangolins, compared to the Asian species, and this difference may be due to the presence of dehydration in some of the animals included in studies concerning the latter (13, 15). Although these Chinese and Sunda pangolins were not obviously dehydrated on examination, in two studies they were sampled around 24 h after being captured from the wild or rescued and may have had decreased access to water (15). The clinical changes expected for different degrees of dehydration in pangolins has not been documented to our knowledge, and mild dehydration may not be detectable on clinical examination. An alternative hypothesis is that Asian pangolins simply have higher concentrations of these measurands, which could be due to differences in diet and protein metabolism.

Bilirubin upper reference limits in the Asian pangolins were as high as 22 μmol/L in Sunda pangolins and 31 μmol/L in Chinese pangolins, compared to almost half those concentrations in Temminck's pangolins (13, 15). Bilirubin is a hemoglobin break-down product, and is taken up from the blood by hepatocytes, conjugated, and excreted into the gastrointestinal tract in bile. Pathological reasons for hyperbilirubinaemia include hemolysis, cholangiohepatopathy and obstruction in the biliary tree or gall bladder (44). It is unlikely that these diseases would have gone unnoticed in a significant proportion of apparently healthy Asian pangolins. In some species, like horses, an increase in bilirubin is seen within 24 h of the onset of fasting or anorexia (44). Pangolins may have experienced anorexia in the period leading up to blood collection in the Sunda pangolin and one of the Chinese pangolin studies, where pangolins were either rescued or captured from the wild and kept in a novel captive environment for around 24 h before blood collection (13, 15). However, the other Chinese pangolin study used animals that were kept captive and fed daily, long-term, and would not have experienced a fasting hyperbilirubinemia (14). It may therefore be the case that healthy Asian pangolins have higher concentrations of bilirubin than Temminck's pangolins. Bilirubin is also quickly degraded by UV light, but sample handling in all studies appears to have been similar, with tubes not unduly exposed to daylight (44).

Juvenile animals have higher ALP activities and phosphate concentrations compared to adults, due to bone metabolism (44). This has been shown in juvenile Sunda and Chinese pangolins, and the high proportion of juveniles in the Asian pangolin studies is likely the reason for the high reference intervals for these two measurands in those pangolins., compared to our Temminck's pangolin group, which only contained a much lower proportion of juveniles (5/27) (13–15). The high upper reference limits for glucose in all studies is likely due to a catecholamine-induced hyperglycemia related to capture and anesthesia (15). Glucose concentrations in captive anesthetized white-bellied pangolins (6.1–8.4 mmol/L) were significantly higher than glucose concentrations measured in blood samples taken from the same animals using manual restraint (4.6–6.9 mmol/L) (16).

Although the reference individuals originated from two groups of Temminck's pangolins living under different husbandry conditions, and significant differences were found for some measurands between the two groups, we did not perform partitioning or generate further reference intervals for the sub-groups. Partitioning is not recommended when resulting in sub-groups of *n* < 40, and sub-group sizes here would have been *n* = 18 (free-living) and *n* = 9 (rehabilitated) (20). Furthermore, it would not have been possible to generate reference intervals for such small groups. Most of the differences between the two groups, in particular the higher sodium, erythron and protein measurands in the free-living pangolins, are most likely related to differences in hydration status, and possibly nutrition. The free-living pangolins were from an arid habitat, and differences in hydration status may be responsible for the relative hemoconcentration and higher protein and sodium levels in this group (45, 46). Splenic contraction secondary to adrenaline release can also result in hemoconcentration in some species, and excitement during capture may play a role here too, as the rehabilitated animals may have been habituated to handling (46). The reasons behind the higher ALT and amylase in the rehabilitated group are unclear. The comparatively higher activities of ALT in the rehabilitated animals may represent differences in protein metabolism (as the function of ALT is the deamination of alanine to pyruvate), hepatocyte integrity, or induction (47). In dogs, ALT may be induced by anesthetic agents and corticosteroids; it is not known whether these substances induce ALT in pangolins. Several isoenzymes of amylase occur in other species (salivary, pancreatic, intestinal and macroamylase) but information for pangolins is lacking (47). The higher amylase activity in the rehabilitated group could represent a relative increase in any of these isoenzymes and should not be over-interpreted as representing pathology. Further investigations as to gastrointestinal, hepatic and pancreatic physiology in Temminck's pangolins are needed.

There were several limitations to this study. The small sample size and use of non-validated methods have been discussed. Because so few animals were included, partitioning, for example for sex or origin, was not performed (20). Another limitation is that the plasma samples were stored for 90 days−24 months before analysis. The clinical chemistry measurands evaluated in our study have been shown to be stable in human serum samples stored at −80°C for up to 10 years, but the effect of storage on heparin plasma, specifically from pangolins, is not known (48, 49). It would also have been beneficial to measure additional measurands not included in the rotor we used, like aspartate aminotransferase, creatinine kinase, cholesterol and triglycerides.

## Conclusion

The reference intervals presented here will prove a useful tool for clinicians working with Temminck's pangolin. The acquisition of blood samples in these animals is fairly easy, and clinical pathology is one of the less invasive diagnostic modalities used in veterinary medicine. Portable point-of-care analyzers, like those used in this study, are particularly convenient for veterinarians working with wildlife, either for research purposes or in rehabilitation settings. Reference intervals do not only have utility in diagnosing and monitoring disease but can also provide information as to the unique physiology of a species. Several interesting differences between this species and Asian pangolins were noted and should be further investigated. Very little veterinary research had been performed on this species so far, and this study should provide a basis for further investigations.

## Data Availability Statement

The original contributions presented in the study are included in the article/Supplementary Material, further inquiries can be directed to the corresponding author/s.

## Ethics Statement

The animal study was reviewed and approved by Animal Ethics Committee of the University of Pretoria. Written informed consent was obtained from the owners for the participation of their animals in this study.

## Author Contributions

EH contributed to conceptualization and study design, sample analysis, data curation, performed the data analysis, took the photomicrographs, acquired funding, and wrote the first draft of the manuscript. KL contributed to conceptualization and design of the study, sample acquisition, sample analysis, data curation, acquired funding, and co-wrote the first draft of the manuscript. LM contributed to conceptualization and study design, sample acquisition, sample analysis, acquired funding, and contributed to the first draft of the manuscript. All authors contributed to manuscript revision and read and approved the submitted version.

## Conflict of Interest

The authors declare that the research was conducted in the absence of any commercial or financial relationships that could be construed as a potential conflict of interest. The reviewer DP declared a shared affiliation with the authors, to the handling editor at time of review.
